# A public health approach to mobilizing community partners for injury prevention: A scoping review

**DOI:** 10.1371/journal.pone.0210734

**Published:** 2019-01-22

**Authors:** Alexander M. Crizzle, Cathy Dykeman, Sarah Laberge, Ann MacLeod, Ellen Olsen-Lynch, France Brunet, Angela Andrews

**Affiliations:** 1 School of Public Health, University of Saskatchewan, Saskatoon, Saskatchewan, Canada; 2 School of Public Health and Health Systems, University of Waterloo, Waterloo, Ontario, Canada; 3 Halton Region Health Department, Oakville, Ontario, Canada; 4 Trent Fleming School of Nursing, Trent University, Peterborough, Ontario, Canada; 5 Eastern Ontario Health Unit, Cornwall, Ontario, Canada; 6 Haliburton, Kawartha, Pine Ridge District Health Unit, Port Hope, Ontario, Canada; Emory University, School of Public Health, UNITED STATES

## Abstract

**Objectives:**

Reducing injuries in adults requires work with diverse stakeholders across many sectors and at multiple levels. At the local level, public health professionals need to effectively bring together, facilitate, and support community partners to initiate evidence-based efforts. However, there has been no formal review of the literature to inform how these professionals can best create action among community partners to address injuries in adults. Thus, this scoping review aims to identify theories, models or frameworks that are applicable to a community-based approach to injury prevention.

**Methods:**

Searches of scientific and less formal literature identified 13,756 relevant items published in the English language between 2000 and 2016 in North America, Europe and Australia. After screening and review, 10 publications were included that (1) identified a theory, framework or model related to mobilizing partners; and (2) referred to community-based adult injury prevention

**Results:**

Findings show that use of theories, frameworks and models in community-based injury prevention programs is rare and often undocumented. One theory and various conceptual models and frameworks exist for mobilizing partners to jointly prevent injuries; however, there are few evaluations of the processes to create community action.

**Conclusions:**

Successful community-based injury prevention must build on what is already understood about creating partnership action. Evaluating local public health professional injury prevention practice based on available theories, models and frameworks will identify successes and challenges to inform process improvements. We propose a logic model to more specifically guide and evaluate how public health can work locally with community partners.

## Introduction

Unintentional injuries, such as those from falls, motor vehicle collisions, poison, drowning, and burns, kill more than 10,000 Canadians each year [1]. Although the vast majority of unintentional injuries are predictable and preventable, the direct (i.e. healthcare) and indirect (e.g., losses in paid productivity due to hospitalization, disability, death) costs to Canadians are over $22 billion annually [2]. To reduce these costs, injury prevention initiatives must address the underlying social, behavioural and environmental determinants through coordinated, community-wide, and multi-sectored strategies [3].

Two approaches have been useful in preventing injuries: universal and targeted. Universal approaches, such as national building codes for stairs, provincial safety standards for motor vehicles, and pool fencing bylaws in municipalities, improve cause-specific safety for the population by addressing injury determinants broadly [4]. Targeted approaches include equity-based initiatives, such as affordable barrier-free housing for people living with disabilities and low-cost safety equipment for those living within lower incomes, address community-specific determinants such as susceptibility or affluence [4] by increasing safety behaviours and ensuring that strategic initiatives are dedicated towards the intended community members [5, 6]. One common feature of both universal and targeted approaches is the need for partnerships across many sectors and levels to address the significant determinants of injury [4]. Although partnership structures may range from the informal to the legally bound, all are working together for the purpose of joint action to achieve a common goal [1].

Our prior projects examined local partnerships between Public Health Units in Ontario and community services addressing falls in older adults, including both health (e.g. home health and health care agencies) and non-health (e.g. social support, recreation, and business) sectors. The findings show that the community partners had insufficient resources to implement fall prevention strategies alone (e.g. exercise programs, medication reviews), despite awareness, intention, and organizational support [7]. Additionally, these community service providers reported multi-level challenges when implementing fall prevention programs, from their geography and service systems, to the specific organizations and the individuals that work within them [8]. However, community partners reported that effective leadership, administration and management by public health professionals was a defining strength of community-based fall prevention initiatives and helped the community to bridge individual resource gaps [9]. These findings confirm that partnerships can build and extend capacity to deliver interventions suited to the local context, effectively pooling the resources, talents, and strategies of diverse community partners [10, 11]. Community-owned solutions rely on partnerships such as those among community members, organizations, public health workers and local governments [12].

Ontario Public Health Standards for injury prevention [13] require working in collaboration with community partners in order to optimize the delivery of public health services by sharing resources and responsibilities [1]. Effective injury prevention requires this concerted effort to formulate context-sensitive and sustainable solutions from published interventions that are typically tested in controlled settings [14]. A recent literature review that examined intersectoral partnerships within health promotion described positive processes for improving population health more generally [15]. How diverse community partners become mobilized to achieve reductions in unintentional injuries remains unclear [9]. While listing promising programs, Ontario’s “Prevention of Injury Guidance Document” currently presents no theories, frameworks or models for how local public health professionals can mobilize community partners in effective injury prevention [13]. Proven interventions must have adequate support to be effective, thus, local public health professionals must also understand how to facilitate community-wide partnerships to produce socially significant injury reductions [16].

Thus, the purpose of this paper was to conduct a scoping review to identify theories, frameworks, and models (TFM) that clarify how to mobilize partners in community-based adult injury prevention and to what effect, seeking evidence from a range of real-world sources rather than being limited to any particular quality standard (e.g. randomized controlled trials). Given there are no published literature reviews, a scoping review is a systematic way to scan and aggregate existing literature to further articulate the concept of mobilizing partners, identify knowledge gaps and inform future directions related to community-based adult injury prevention.

## Methods

### Project team

This project involved a multidisciplinary team including nine public health professionals from Ontario public health units, two academics, an academic librarian, a graduate student, and a research consultant, with collective expertise in injury prevention, scoping reviews and knowledge translation activities. Member engagement varied, with two project leads managing the collaboration, the consultant coordinating the research project, 6 team members reviewing literature, and knowledge users providing stakeholder advice. All members participated in bi-weekly teleconferences, building relationships to integrate knowledge translation between researchers and practitioners. In addition, three face-to-face meetings actively involved end-users at the various stages of the research, with each contributing their own distinct expertise and experience to the process.

### Identification of relevant studies

A methodological framework, developed by prior authors, guided our scoping review process [17, 18]. The first step entailed defining the working terms (see Table 1).

**Table 1:** Working definitions for search terminology.

Term	Definition
Theories	Systematic views of interrelated concepts, definitions, and propositions to explain and predict events or situations [19].
Frameworks	Broad structures of descriptive categories containing both explicit and assumed propositions [20].
Models	Symbolic representations of theoretical concepts for further understanding of a problem in a specific context [21, 22].
Mobilization	Moving a group of people into collective action.
Community Partners	Organizations with a vested interest in injury prevention or an interest in the well-being of adults living independently with some common characteristic, for example locale, faith or heritage. Living independently includes in group homes and the homeless but not those in institutions such as hospitals, treatment facilities, long term care homes, jails, or similar. For our purposes community partners may include governmental agencies, non-governmental agencies, coalitions, networks, industry, employers, location-specific organizations, health authorities, healthcare institutions, etc. Citizens were not included as partners to be mobilized but rather as clients of public health services and drivers of any improvements to their health and wellbeing.
Community-based Adult Injury Prevention	Reduction of the frequency, severity and impact of injury among adults (18+) through community involvement. The community-based injury prevention model emphasises that community participation and multidisciplinary collaboration are needed to address local problems [23].

Our literature search included documents of any study design and publication type. Articles were selected based on the following inclusion criteria: 1) published between 2000 and 2016; 2) published in the English language; 3) related to community-based injury prevention among community dwelling adults; 4) presenting one of a TFM for mobilizing community partners; and 5) based on literature in developed countries similar to Canada such as the United States, Australia, New Zealand and high income European nations. We excluded articles that were 1) book reviews, editorials or commentaries; 2) not in a developed high income country; 3) without any TFM for mobilizing community partners; 4) not related to community-based injury prevention; and 5) not addressing an adult population of 18 years and older.

The librarian member developed search strategies to identify relevant articles in consultation with Public Health Ontario researchers and Shared Library Services. Subject headings and keywords included: 1) theory/framework/model (e.g., paradigm, strategy, approach); 2) mobilization (e.g., capacity building, coalition, collaboration, community network, multi-sector partnership); and 3) injury prevention (e.g., accident prevention, falls, protective devices, safety). The first search stage included databases such as Ovid MEDLINE (including In-Process & Other Non-Indexed Citations and Epub Ahead of Print), EBSCO CINAHL, ProQuest PsycINFO, ProQuest Sociological Abstracts. The second stage of searching included Theses Canada, DART Europe E-theses Portal, EThOS (UK e-Theses online), TROVE (National Library of Australia), Public Health Databases and Grey Literature Repositories (Canadian Public Policy Collection, Canadian Best Practice Portal/Public Health Agency of Canada, Ontario Prevention Clearinghouse), Custom Search Engines (Canadian Public Health Information, Ontario Public Health Units, US State Government Information). The third stage of searching consisted of websites suggested by project team members (Agency for Healthcare Research & Quality, Health Canada, National Collaborating Centre for Methods and Tools, Centre for Disease Control, NICE UK, Robert Wood Johnson Foundation, Tamarack Community, Art of Hosting, Google). Hand searches were also performed using 1) the reference lists of the included items; 2) the tables of contents of three journals that publish articles related to injury prevention and public health (e.g. Health Promotion Practice, Health Promotion Journal of Australia, American Journal of Public Health); 3) additional suggestions made by project team members; and 4) publications of eight well-known authors in the field of community health partnerships identified by our academics (i.e., Neil Bracht, Francis Butterfoss, Stephen Fawcett, Paul Florin, Michelle Kegler, Meredith Minkler, Nina Wallerstein, and Abraham Wandersman).

### Study selection

**Fig 1:**
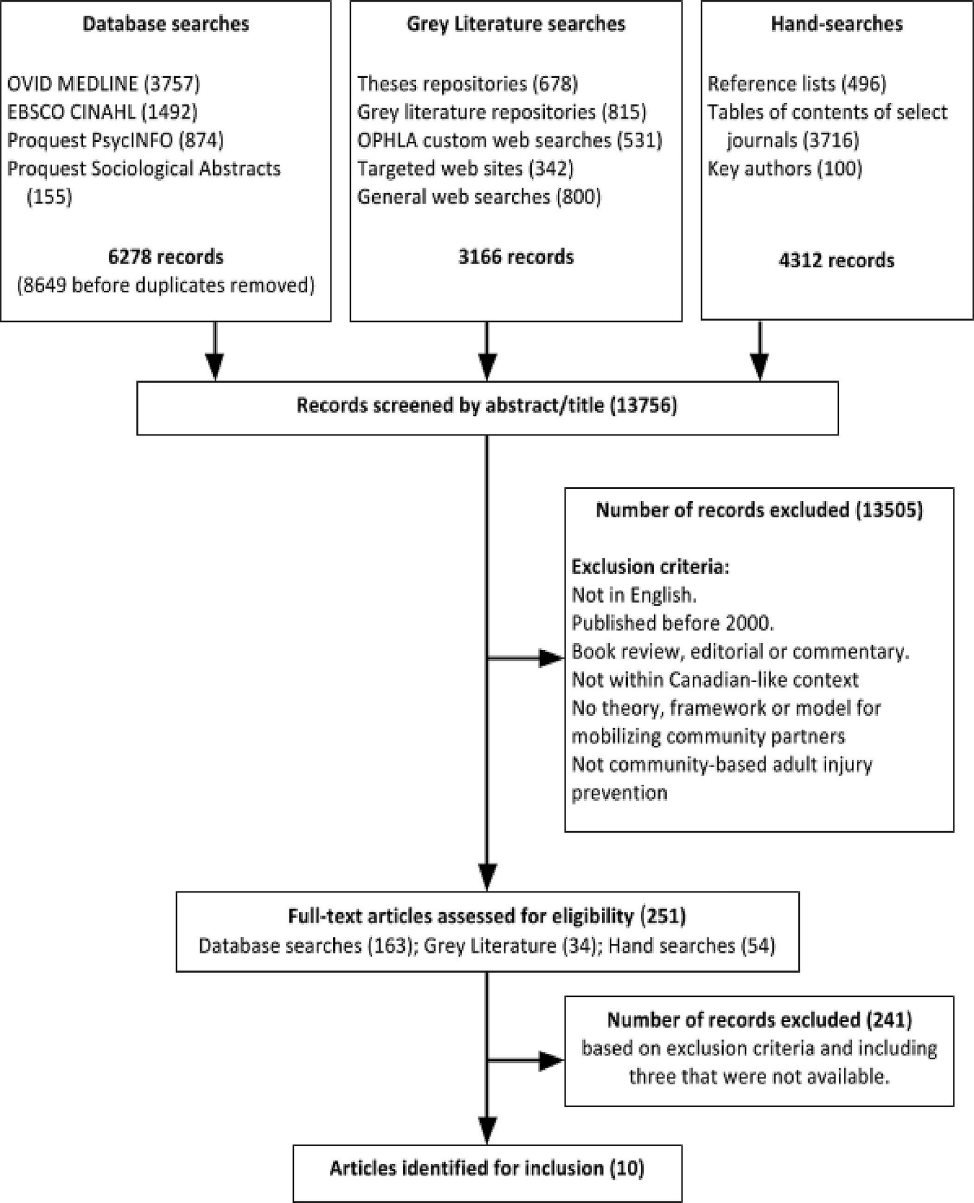
Flow diagram for scoping review process.

As shown in **Fig 1**, the initial search yielded 13,756 sources of literature. A database management system (EndNote) removed 2371 duplicates. We established a reviewer group that included the research consultant, an academic, a graduate student, and three public health nurses. We calibrated consistency in determining study inclusion and exclusion was among all reviewers, first through independent assessments (yes, no, unsure) of 25 random abstracts followed by paired assessments of 75 random abstracts. Reviewers agreed on 83% of the abstracts to be included. We discussed and agreed upon differences via consensus. Following these exercises, we distributed all titles and abstracts among the reviewer group to screen for inclusion by asking two questions: “Does the title or abstract identify a theory, framework or model related to mobilizing community partners?” and “Does it refer to community-based adult injury prevention?” Reviewers excluded all but 251 items and these advanced for a more in-depth review of their full text. We then distributed full-text articles among the reviewer group to determine further exclusions using the screening criteria as well as an additional criteria “Is the context of development and/or implementation of models, theories and frameworks in a developed country similar to Canada (e.g. United States, Australia, New Zealand, high income European nations)”, which may not have been apparent from the title or abstract alone. Any full text articles rated as “unsure” by the first reviewer were assigned to a second reviewer and discussion determined final eligibility for inclusion. Reviewers presented the 10 publications that satisfied our criteria for inclusion to the full project team to verify perceptions of relevance to our research question and applicability within public health practice, as well as for knowledge exchange.

### Data charting, collating, summarizing, and reporting

A data extraction tool captured the reference source, literature type, study design, and the identified TFM and incorporated a critical appraisal tool for the included literature. The MetaQAT or the Public Health Ontario Meta-tool for Quality Appraisal of Public Health Evidence [24] is a novel critical assessment tool developed to examine article quality (i.e. relevancy to our research question, applicability to public health unit practices in Ontario, reliability of design and validity of results). Using one of the included articles, project team members (consultant, student and public health nurse) independently tested the data extraction tool for clarity and ease of use. The MetaQAT does not provide a numerical score for rating articles; rather, long-form responses recorded the identified strengths and weakness of each literature source. At least two project team members appraised and summarized the ten included articles.

As not all included articles provided complete descriptions of the identified TFM, supplementary sources provided the full list of key elements comprising each TFM (e.g., constructs, steps, components, phases, tasks). Two project team members (CD, SL) pragmatically sorted the elements of each TFM into three broad categories related to 1) prerequisite conditions for community partner activity (e.g. formation as a group), 2) results of community partner activity (e.g. achievements), and 3) neither prerequisite or resultant, thereby distinguishing transitional elements to conceptualize partner mobilization. Subsequently, the full multidisciplinary team reviewed, refined and confirmed the associated categories.

## Results

The ten sources collectively identified one theory, seven frameworks, and six models for mobilizing partners. Three publications identified more than one framework or model [11, 25, 26]. Five of the sources were specifically set in an injury prevention context [23, 26–29] with two focussed on generally preventing injury across all ages [23, 26], two focussed on preventing recreational and professional sport injury among adult men aged 18 to 35 years old [27, 28] and one focussed on preventing fall-related injury among adults 65 years of age and older [29]. The remaining five sources addressed adult injury prevention more generally as part of population health improvement initiatives [11, 25, 30–32]. Five sources provided brief descriptions of a TFM [11, 25, 28, 30, 32], four sources proposed a new framework or model [23, 26, 29, 31] and three described applications [25, 27, 28].

### Characteristics of the included literature

Table 2 summarizes the included sources based on the relevance to the research question, applicability to local public health agencies, reliability (or reproducibility) and validity (rigor of research). Four of the ten sources were primary articles including two case studies and two were quasi-experimental (repeated measures) design. One case study applied Step 5 of the Intervention Mapping Framework [33] to prevent Australian football injuries in adult males [27]. The second case study presented 12 core components across four stages of coalition building to propose a Coalition Development Model for successful collaboration [26]. One study used a repeated measures design to evaluate a multi-agency partnership between the government (health promotion division), safety agencies, sports professionals, advocacy bodies and health insurance organizations to reduce recreational sports injuries over a 5-year period [28]. Another study used a repeated measures design to monitor physical activity programming intended to reduce falls and related injuries among older adults, reporting a process and impact evaluation of the Collaborative Management model that supported a promotional campaign [29].

**Table 2:** Characteristics and quality of literature sources.

Author, Publication Year, Location	Document Type	Context	Relevance to research question, applicability within the scope of Ontario public health units, reliability and validity (MetaQAT)	Identified Theory, Framework, and Models related to mobilizing partners
Batan et al. [25] USA	Secondary: lay summary of evidence	Communities funded by Healthy Communities Program (CDC)	**Relevant**: guides coalitions to establish, advance, and maintain effective strategies that improve community health (including injury prevention) **Applicable**: developed for coalitions and public health professionals with steps and learning modules; based on Healthy Communities initiatives that could be applied in Ontario settings **Reliable**: extensively-referenced practice-based support by multidisciplinary, multi-level, multisector writers and consultations in 300 communities **Valid**: biased by positive examples only (no failures included) from communities across the US	• Community Coalition Action Theory [34] Model of Sustainability Approaches [35] • Framework for fostering productive collaborations and coalitions • Framework for building capacity for operational purposes
Donaldson et al. [27] Australia	Primary: case study	Multi-agency partnership to address sport-related injury prevention	**Relevant**: protocol for injury prevention program planning focusing on adoption, implementation and evaluation within diverse community groups **Applicable**: knowledge, skills and experience of diverse groups are valued in theory and evidence informed planning for adoption, implementation and evaluation of health promotion projects **Reliable**: step by step, evidence-informed implementation planning protocol; authors claim it to be a practical, useful and scalable process to planning the implementation of a tested health promotion intervention **Valid**: unclear how claims of high quality planning, efficient, easy-to-use, improved fit were assessed; claim of transferability to “any context” is not supported by study design but protocol has been used successfully in multiple contexts	• Intervention mapping [33]
Downey et al. [26] USA	Primary: case study	Four rural injury prevention coalitions	**Relevant**: provides indicators of essential components for injury prevention across each of the four developmental stages of coalition development **Applicable**: key components and processes for perceived success by four, rurally situated U.S. safety coalitions were identified; authors claim the pathway can guide public health practitioners in supporting coalitions through all phases. **Reliable**: member checking done to ensure accurate data analysis and findings **Valid**: Document analysis of coalitions, media coverage, and cross- sectional survey results were analysed by researchers and verified with coalition members.	• Model of Coalition Development • Theory of Coalitions [36]
Fawcett et al. [30] USA	Secondary: literature review	Multi-sectoral partnerships to achieve population health goals	**Relevant**: generic model for collaborative action to improve health of communities **Applicable**: Seven recommendations for strengthening collaborative partnerships for population health and health equity were proposed by authors **Reliable**: no methodology described **Valid**: methodology for synthesis was not described; merit relies on expert opinion	• Framework and Processes for Collaborative Action: 5 key components and 12 key processes [37]
Finch et al. [28] Australia	Primary: repeated measures study	Multi-agency partnership to address sport-related injury prevention	**Relevant**: provides evaluation framework of partnership effectiveness **Applicable**: objective unclear as other than to show that the partnership has changed, there is no assessment of why it changed **Reliable**: the repeated measures design was appropriate; information on the measurement and results are adequate for reproducibility **Valid**: Partnership evaluation tool has not been validated	• Partnership Continuum Framework [38]
Florida Department of Health [32] USA	Tertiary: brochure promoting strategic planning tool	Community health assessment and improvement planning	**Relevant**: generic framework for Local Health Department engagement in community health improvement activities; has been used in the injury prevention field; **Applicable**: generic process framework has been used to secure government funding by public health led coalitions that address community health improvement **Reliable**: clearly detailed with necessary steps; supplementary roadmap, user handbook, field guide available, used by hundreds of American Local Health Departments **Valid**: more than ¾ of the 200 American Local Health Departments using the framework found it effective, flexible, comprehensive, worth the effort and would recommend it to others.	• Framework for Mobilizing for Action through Planning and Partnerships [39]
Meyers et al. [31] USA	Secondary: scoping review	25 implementation frameworks	**Relevant**: only 6/25 frameworks from community-based initiatives, only one applied to injury and violence prevention, none to adults, and none to fall prevention **Applicable**: the focus is on quality implementation, defined as “putting an innovation into practice in a way that meets the necessary standards to achieve desired outcomes”. Practical steps and specific actions (i.e. the ‘how to’). **Reliable**: no quality assessment of framework **Valid**: systematic methodology reported	• Quality Implementation Framework
Nilsen et al. [23] Sweden	Secondary: literature review	Community-based injury prevention programs	**Relevant**: synthesis specific to community-based injury prevention; program development and evaluation activities **Applicable**: the injury prevention logic model developed by authors propose that it answers not just ‘does it work’ but also ‘how and why it works’ **Reliable**: methodology not described **Valid**: methodology not described	• Community-based injury prevention program logic model
Stackpool et al. [29] Australia	Primary: repeated measures study	Multiple health agency collaboration to increase physical activity programming for fall injury prevention	**Relevant:** evaluation for shared decision-making, resource use and coordination **Applicable:** a multi-level collaborative management structure (networks, health agencies, services) was not appropriate for local implementation, only the regional media campaign development **Reliable:** inadequate information on the methodology; semi-structured interviews with key stakeholders and workshops were appropriate for reviewing the model **Valid:** lack of detail around data analysis of model utility	• Collaborative management model
Woulfe et al. [11] USA	Secondary: literature review	Partnerships for population health improvement	**Relevant:** key factors (goals, sponsorship, resources, membership, leadership) that are associated with effective multi-sector partnerships **Applicable:** key organizational and contextual factors important to effective collaboration among diverse groups addressing population health improvement **Reliable:** limited to mainly participant perceptions rather than objective measures **Valid:** methodology not described	• Typology of partnerships for health improvement adapted [40]

Five of the ten sources were secondary literature: four reviews (three narrative reviews and one scoping review) and one synthesis of evidence for community action. One author presented 5 components and 12 processes to guide communities’ capacity to improve their health based on the Collaborative Public Health Action Framework [30]. Another author presented a typology and factors influencing effectiveness of multi-sector partnerships to improve population health [11]. The third author presented an evidence-informed logic model for community-based injury prevention programming [23]. The scoping review author synthesized 14 critical steps for implementing quality improvement innovations [31]. The synthesis of evidence for community action was the Centre for Disease Control’s Sustainability Planning Guide for Healthy Communities designed to help coalitions, public health professionals and community stakeholders develop, implement and evaluate sustainability plans. [25].

One of the ten sources was tertiary: an online brochure for community health improvement using the model of “Mobilizing for Action through Planning and Partnerships” [32]. Table 3 illustrates the transition from establishing prerequisites to achieving results based on the key elements of the TFMs identified by the included literature.

**Table 3:** Overview of theory, frameworks, models highlighting elements related to mobilizing partners.

	Name of Theory, Framework, Model	Pre-requisites for community partner activity	Transitional elements of partner mobilization			Results of community partner activity
			Member engagement	Instrumental supports	Planned action	
Theory	Community Coalition Action Theory [34, 36]	• Operation and processes • Leadership and staffing • Structure	• Member engagement	• Pooled resources	• Assessment and planning • Implementation of strategies	• Community change (capacity or health outcomes)
Framework	Intervention Mapping [33] https://interventionmapping.com/	• Logic model of the Problem • Outcomes/objectives; Logic model of Change • Program Design • Program Production			• Implementation plan	• Evaluation plan
	Collaborative Public Health Action [37] https://www.ncbi.nlm.nih.gov/books/NBK221228/	• Logic model • Develop and use strategic/action plans • Define organizational structure and operating mechanisms	• Arrange community mobilization	• Develop leadership		• Changed conditions in communities and systems • Widespread change in behaviour and risk factors • Improved population’s health
	Partnership continuum [38]	• Networking • Coordinating	• Collaborating	• Cooperating		
	Quality Implementation Framework [31]	• Assessment • Decisions about adaptation • Capacity-building		• Implementation teams	• Implementation plan	• Technical assistance/coaching/ supervision • Process evaluation • Supportive feedback mechanism • Improve future applications
	Determinants of effective partnerships in health [11]	• Partnership resources • Core resources • Vision • Leadership • Organizational structure • Membership • Quality of relationships • External and contextual factors (e.g. issue, timing, target)	• Leadership • Membership • Quality of relationships • Vision	• Partnership resources • Core resources • Organizational structure		
	Conditions that foster productive collaborations [25]	• Build community ownership. • Assess community needs and assets. • Develop commitment for vision, mission, goals, and objectives. • Create viable organizational structure. • Recruit key organizational members. • Build leadership team	• Retain member commitment and participation to achieve objectives	• Obtain and share member resources	• Plan and implement effective Policies, Services, Environments	• Diversify and strengthen coalition’s financial base • Ensure community Home (i.e. base of operations) for ongoing efforts. • Plan for leadership succession. • Institutionalize strategies within member organizations and community institutions.
	Building operational capacity [25]	• Create a shared understanding of sustainability • Create a plan to work through the process • Position coalition efforts to increase the odds of sustainability Look at the current picture and pending items • Develop criteria to help determine which efforts to continue • Decide what to continue and prioritize • Create options for maintaining priority efforts • Develop a sustainability plan	• Implement the sustainability plan: Keeping people involved		• Implement the sustainability plan: Action plans	• Evaluate outcomes and revise as needed
	Sustainability Framework [25]	• Develop policy, systems, and environmental change strategies. • Build the long-term capacity of your coalition and relevant partnerships to achieve policy goals. Complemented by: • Establish a home for your work. • Focus on building coalition members’ skills. • Develop communication strategies. • Develop social marketing strategies.			• Implement policy, systems, and environmental change strategies • Implement communication strategies • Implement social marketing strategies	
Model	Coalition development model [26]	• Collaborate with numerous partners to obtain funding • Define what data available • Define coalition structure • Recruit members • Notify community • Identify potential partners • Structure and facilitate meetings appropriately • Notify community leaders and businesses • Identify needed education • Link with partners • Build alliances with media • Develop an evaluation plan	• Formalize structure • Empower members • Network with similar groups • Watch for other beneficial partners • Significant roles for active members • Seek community support	• Sustain funding • Collect and analyze data • Channel messages • Reach out beyond • Keep efforts in media • Regularly evaluate process structure		• Regularly evaluate outcomes • Use data to guide coalition efforts • Continually seek new members • Delegate appropriately • Continue expanding efforts • Determine future endeavours • Keep the coalition’s agenda top of minds • Expand to new channels • Identify future opportunities • Communicate the coalition’s agenda in the media • Improve the coalition according to evaluation
	Multifaceted community-based injury prevention program logic model [23]	• Recognition of community health or safety problem • A decision to address the problem • Participatory planning • Sufficient resources (human, relational, structural) to deliver the interventions • Assessment of community variables impacting injury as well as interventions	• Community members and local organizations are involved in the delivery	• Capacity to mobilize depends on level of human, relational and structural resources	• Strategic planning and program operation	• Program exposure • Attitudinal effects • Injury risk effects • Safety effects
	Mobilizing Action through Planning and Partnerships [39] http://www.naccho.org/programs/public-health-infrastructure/map p Community Tool Box http://ctb.ku.edu/en/table-of-contents/overview/models-for-community-health-and-development/mapp/main	• Organize for success & Partnership development • process is organized and planned out • core group and an inclusive steering committee • Vision • 4 MAPP assessments Identify strategic issues • Formulate goals and strategies • Consider real or potential forces of change			Planning Implementation	• Evaluation
	Collaborative Management [29]	N/A	Collaboration between state and local levels were not found to be a suitable approach for the project’s purpose			N/A
	Partnerships for health improvement [11, 40]	• To extend the reach and capacity of governmental public health who has primary responsibility • To deliver public health services with others who play some role in promoting public health. • To address public health through all sectors of community life (eg, education, business) as the system of actors and actions that promote or threaten population health • To create change primarily by impacting the context through the relationships among organizations in the partnership				

**Note:** Each bullet represents an element identified in the theory, framework or model for establishing and maintaining coalitions and partnerships.

### Theory, frameworks and models partnership activities

Table 4 shows the common steps and elements within each theory, framework and model for developing sustainable and working partnerships. The common elements were grouped based on the timing of the partnership process and distinguish prerequisite and resulting conditions from the transitional, and hence “mobilizing”, elements. Prerequisite conditions to mobilizing consist of partnership start-up, community assessment and strategic planning; resulting conditions are centered on evaluation; and ongoing involvement, instrumental supports and planned action describe transitional mobilizing elements.

**Table 4:** Components of partnership mobilization activities of each model, theory and framework mobilization activities of each model, theory and framework.

Source	Batan et al. [25]			Bartholomew et al. [33]	Butterfoss et al. [36]; Butterfoss& Kegler [34]	Downey et al. [26]	Fawcett et al. [30]	Himmelman [38]	Meyers et al. [31]	NACCHO [39]	Nilsen [23]	Stackpool [29]	Wolfe et al. [11]	
	Conditions that foster productive collaborations	Building capacity for operational purposes	Sustainability Approaches	Intervention mapping	Theory of Coalitions, Community Coalition Action Theory	Coalition Development	Collaborative Action	Partnership Continuum	Quality Implement- ation	Mobilizing for Action through Planning and Partnerships	Community-based injury prevention program logic	Collaborative management	Typology of partnerships for health improvement	Determinants for effective public health partnerships
Pre-Mobilization														
	**Partnership Start-Up (13/14 or 93%)**													
United in commitment 12/14 (86%)	x	x			x	x	x	x	x	x	x	x	x	x
Structured encounters 10/14 (71%)	x		x		x	x	x		x	x		x	x	x
Recruitment 8/14 (57%)	x		x		x	x			x	x		x		x
Member development/skill building 6/14 (43%)	x		x		x	x				x				x
	**Community Assessment (12/14 or 86%)**													
Understanding of issue 9/14 (64%)	x	x		x	x		x		x	x	x			x
Resources 10/14 (71%)	x	x	x		x	x		x	x	x	x			x
Participation 10/14 (71%)	x	x	x		x	x		x	x	x	x			x
	**Strategic Planning (9/14 or 64%)**													
Logic model 3/14 (21%)				x			x				x			
Planning, adaptation 9/14 (64%)		x	x	x	x		x		x	x	x	x		
Mobilization														
	**Ongoing Involvement (8/14 or 57%)**													
Continued participation 6/14 (43%)	x	x			x	x		x			x			
Commitment 4/14 (29%)					x	x		x						x
Leadership 3/14 (21%)					x		x							x
Collective aim 2/14 (14%)								x						x
	**Instrumental Supports (8/14 or 57%)**													
Collective resources 8/14 (57%)	x				x	x	x	x	x		x			x
Public accountability 3/14 (21%)					x	x		x						
	**Planned Action (9/14 or 64%)**													
Planning: 8/14 (57%)	x		x	x	x	x			x	x	x			
Implementation 6/14 (43%)	x	x	x		x	x				x				
Post-Mobilization														
	**Evaluation (7/14 or 50%)**													
Evaluation plan 5/14 (36%)		x		x	x	x					x			
Evaluation implementation 3/14 (21%)						x	x			x				
Celebrate / improve / sustain 3/14 (21%)					x	x	x							

#### Pre-requisites to mobilization

Partnership Start-up. All but one TFM noted the importance of developing partnerships. However, the literature had different accounts of what aspects were critical to developing partnerships. United in commitment was most frequently mentioned (86%), followed by structured meetings (71%), recruitment of stakeholders and key organizational members and partners (57%) and skill building activities (43%).

Community Assessment. Twelve of the TFM noted the importance of performing community assessment, defined as determining priority areas for intervention, as part of building productive partnerships. Most noted understanding the issue or problem, and the sharing of resources and active participation (planning, meetings, skill building), 64% and 71% respectively, in all the TFM’s.

Strategic Planning. Strategic planning consists of determining how an intervention developed through partnership work will result in improved outcomes. While 64% found that planning and adaptation (developing strategic plans, action plans, interventions, policies) was an important pre-mobilization activity, only 21% of the TFMs mentioned the need for a logic model to guide programs and services.

#### Transition to mobilization

Ongoing involvement. More than half (57%) of the TFM identified ongoing involvement with community members, partnerships and stakeholders, categorized as participation (43%), commitment (29%), leadership (21%), and collective aim (14%). One theory, three frameworks, and two models identified member participation. One theory, two frameworks, and one model identified member commitment (29%) as engagement, empowerment, and high quality, trusting relationships.

Instrumental supports. More than half (57%) of the TFMs identified instrumental supports, categorized as collective resources (57%) and public support (21%). One theory, five frameworks, two models identified collective resources, including material resources such as data and sustained funding, human resources such as a designated implementation team, and relational resources such as additional partners outside the partnership that can help with mobilization efforts with the greater community. These resources among core members or across the broader partnership should be shared for mutual benefit and common purpose. One theory, one framework, and one model identified public accountability, including context and community support that could be generated generically through grassroots development and community organizing or specifically through messaging, networking, reaching out and staying visible in the media.

Planned Action. More than half (64%) of the TFMs identified planned action, categorized as planning (57%) and implementation (43%). One theory, four frameworks, three models identified planning, as designing outputs such as effective policies, services and environments, designing processes such as strategies for change, communication, and social marketing, and designing actions such as implementation and program operation. One theory, two frameworks, three models identified implementation related to what was designed: effective policies, services and environments, strategies, actions, a sustainability plan, program operation (e.g. implementation).

#### Mobilization Results

Evaluation. Post-mobilization activities consist of evaluating to determine whether an intervention, program or service has had positive effects on the population of interest. Only 50% of the TFMs identified evaluation activities post mobilization including carrying out evaluations plans (36%), evaluating the implementation of a program or service (21%) and sustaining service delivery (21%).

## Discussion

Although none of the TFM includes all the components necessary for forming and sustaining productive partnerships [23], the findings show that there are common features among the TFMs. These common features suggest that there are preconditions and transitional elements needed to achieve results, thereby contributing to the overall understanding of what successful mobilization should encompass. While each of the TFMs offer conceptual insights into partner mobilization, they independently lack detailed evidence needed to inform, support and guide public health practitioners who are mandated to advance community-based adult injury prevention.

**Fig 2:**
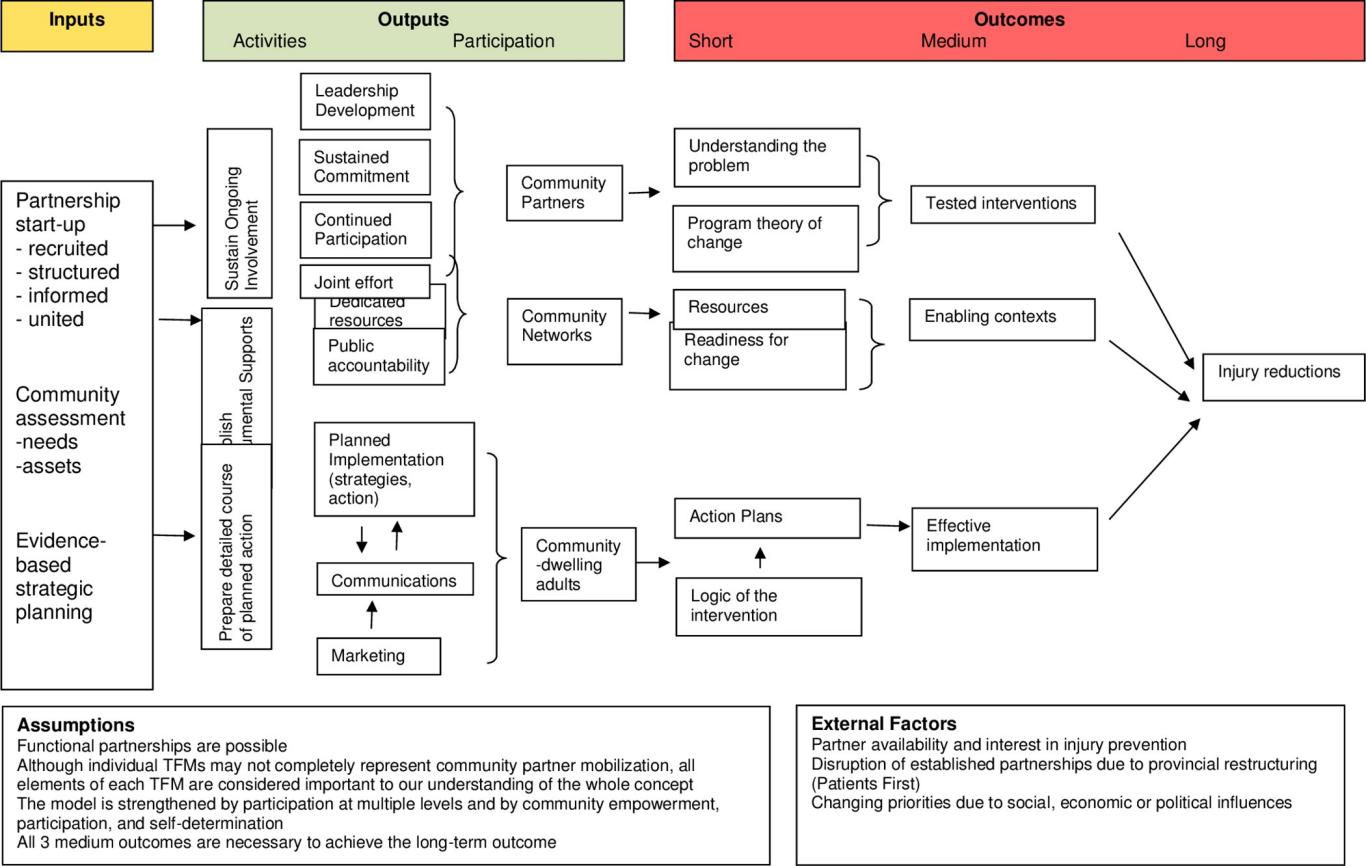
Logic model: Mobilizing community partners in injury prevention.

Our findings also suggest there is less attention dedicated to maintaining and evaluating community-based adult injury prevention work, particularly in the mobilization and post-mobilization phases. We found only one process or outcome evaluation among the studies reviewed indicating a lack of evidence to support investment in such partnership work similar to a prior review that found that behavioural and social science theories and models are seldom represented within injury prevention research [41]. Although the logic model by Nilsen [23] provides theoretical considerations for what works, how and why, very few studies we reviewed included an assessment of reliability and validity. More than half of the reviewed documents did not provide information on the methodologies used to construct their respective TFM. Therefore, when considering all of the TFMs in this review that included the need for evaluation, we developed a logic model that outlines the required inputs, outputs and desired outcomes related to injury prevention initiatives in community settings (see **Fig 2**) to articulate what a partnership effort needs, what it expects to do, how it will work and what success it hopes to achieve.

The prerequisite conditions for community-based injury prevention initiatives form the logic model inputs, and include partnership set-up, community assessment, and evidence-based strategic planning. Involvement from the outset can result in trust building, membership, and ownership among partners for better program implementation [42]. Of the TFMs found in our review, only 57% included ongoing involvement suggesting that many current TFMs perhaps do not reflect the importance of involving stakeholders and organizations throughout program planning and implementation as central in a community organization approach [43].

Partner mobilization forms the logic model outputs, and includes ongoing involvement, instrumental supports, and a detailed course of action, within an ecological framework of the community organizations and networks that can contribute to the context for injury and its prevention among community-dwelling adults. Application of the ecological model in injury prevention holds the most promise in regard to fall and traffic injury prevention, as well as community safety promotion programs [44]. Results form the logic model outcomes, and include building awareness and knowledge of evidence-based interventions, creating enabling environments, and effective community action to achieve injury reductions. A health promotion approach is particularly useful for injury prevention because it specifically facilitates both behavioural and environmental change [45].

Our logic model is consistent with a recent literature review of the components for developing and reinforcing positive partnerships. Partnership start-up includes shared mission, trust and shared power, clarity of roles and structure and appropriate communication. Sustained active involvement includes collaborative leadership. Instrumental supports include active contribution of financial and human resources and consideration of context. A prepared course of action balances maintenance and production. The logic framework format supports evaluation of outputs and outcomes [15].

Mobilizing partners requires attention to sustaining their involvement, obtaining the resources needed (including time and knowledge), and having a clear course of action acceptable to the adult community. In an evaluation of the Healthy Communities movement, findings show that initiatives often rely on community volunteerism and lack the well-supported infrastructure that is essential to successfully maintain ongoing partner involvement [46]. Instrumental supports may come directly from partnering organizations or from the connections and relationships each partner has within the community (i.e. community networks), thereby establishing an enabling context by providing the resources and visibility needed. Such accountability is foundational to mutual trust between public health and communities for building the community’s capacity to address injuries, consistent with findings from a prior review [15]. Communicating and delivering detailed actions keeps implementation transparent and timely, as instrumental supports (i.e. funding, time, accountability, using data to inform policy and practice) may otherwise subside in practice. Thus, it can be seen that collaborative participation in making change, so valued in public health, will lead to program implementation, evaluation and knowledge translation activities that will result in a reduction of injuries.

While it is clear that evaluation-based activities are needed to assess short and long-term outcomes of partnership development, measuring the success of both partnership formation and project management is essential to injury prevention initiatives. This model provides a framework for fully measuring the success of building productive partnerships with definitive outcomes related to injury prevention.

Evidence from measuring the success of community-based partnership development and resulting action is required to justify further public health expenditures. Without literature evaluating community-based adult injury prevention through partnership, public health practitioners will continue to struggle to mobilize and evaluate partnership work with reduced resources. Under these conditions, it is not surprising that intersectoral collaboration is a challenge to maintain, particularly as other non-profit organizations, businesses and health care providers also need evidence to justify investment as a community partner. Now more than ever, community partnerships and local based programs are needed given the high financial strain on the health care system from unnecessary injury related hospital visits. Future work in this field requires a greater emphasis on measuring short and long-term outcomes related to community-based injury prevention initiatives, as well as public health efforts to collaborate with multi-sector partners, to enhance both the reliability and validity of such mobilization and programmatic efforts.

### Study limitations

Although our team members followed a collaboratively developed and refined the search protocol (inclusion and exclusion criteria), it is possible that we missed some sources of literature, particularly as we searched through multiple large bodies of sources. Additionally, we may have missed sources of literature published prior to 2000. We also did not provide evidence ratings of each included article, however, we did provide qualitative feedback using the MetaQAT. This alone should help in assessing reliability and validity, and evidence ratings could be a valued contribution when a more guided systematic review is performed in the future.

## Conclusion

In summary, this scoping review synopsized the theories, frameworks and models (TFM) related to community-based injury prevention found in the available literature. We propose a logic model that combines important components from all the included TFMs into a promising process that better underscores the inputs and activities needed for public health practitioners to build and sustain partnerships active in preventing injuries, as well as plan for evaluating their success.
